# No Additional Prognostic Value of Genetic Information in the Prediction of Vascular Events after Cerebral Ischemia of Arterial Origin: The PROMISe Study

**DOI:** 10.1371/journal.pone.0119203

**Published:** 2015-04-23

**Authors:** Sefanja Achterberg, L. Jaap Kappelle, Paul I. W. de Bakker, Matthew Traylor, Ale Algra, SMART Study Group and the METASTROKE Consortium

**Affiliations:** 1 Department of Neurology and Neurosurgery, Utrecht Stroke Center, Brain Center Rudolf Magnus, University Medical Center Utrecht, Utrecht, The Netherlands; 2 Department of Epidemiology, Julius Center for Health Sciences and Primary Care, University Medical Center Utrecht, Utrecht, The Netherlands; 3 Department of Medical Genetics, Center for Molecular Medicine, University Medical Center Utrecht, Utrecht, The Netherlands; 4 Clinical Neurosciences, University of Cambridge, Cambridge, CB2 0QQ, United Kingdom; University of Münster, GERMANY

## Abstract

**Background:**

Patients who have suffered from cerebral ischemia have a high risk of recurrent vascular events. Predictive models based on classical risk factors typically have limited prognostic value. Given that cerebral ischemia has a heritable component, genetic information might improve performance of these risk models. Our aim was to develop and compare two models: one containing traditional vascular risk factors, the other also including genetic information.

**Methods and Results:**

We studied 1020 patients with cerebral ischemia and genotyped them with the Illumina Immunochip. Median follow-up time was 6.5 years; the annual incidence of new ischemic events (primary outcome, n=198) was 3.0%. The prognostic model based on classical vascular risk factors had an area under the receiver operating characteristics curve (AUC-ROC) of 0.65 (95% confidence interval 0.61-0.69). When we added a genetic risk score based on prioritized SNPs from a genome-wide association study of ischemic stroke (using summary statistics from the METASTROKE study which included 12389 cases and 62004 controls), the AUC-ROC remained the same. Similar results were found for the secondary outcome ischemic stroke.

**Conclusions:**

We found no additional value of genetic information in a prognostic model for the risk of ischemic events in patients with cerebral ischemia of arterial origin. This is consistent with a complex, polygenic architecture, where many genes of weak effect likely act in concert to influence the heritable risk of an individual to develop (recurrent) vascular events. At present, genetic information cannot help clinicians to distinguish patients at high risk for recurrent vascular events.

## Introduction

Patients who suffered from cerebral ischemia have an increased (long-term) risk of new cerebrovascular and cardiovascular events. The American Heart Association recommends the Framingham risk score as a prediction model for major vascular events.[1, 2] Recently this model was compared with six other models in terms of calibration and discrimination.[3] Almost all models slightly overestimated the risk for major events in low and high risk patients. Addition of genetic information might improve these models, but so far this has not yet been evaluated.

Several studies have been performed with a candidate gene approach to find an association between single nucleotide polymorphisms (SNPs) and first ischemic strokes, with often conflicting results, possibly due to small sample sizes in earlier studies and heterogeneity of the stroke subtypes. Two loci (*PITX2* and *ZFHX3*) were found to be associated with atrial fibrillation and cardioembolic stroke risk.[4, 5] Subsequently, two loci (9p21 and *HDAC9*) were identified as robust associations with large vessel stroke.[6, 7] These findings were recently confirmed in a meta-analysis of genome-wide association studies (GWAS) carried out by the METASTROKE consortium.[8] These common genetic polymorphisms account only for a small increase in disease risk, suggesting that large sample sizes will be needed to find additional susceptibility alleles.[9]

Associations between genetic polymorphisms and recurrence of vascular events following an initial episode of cerebral ischemia have received little attention. The aim of our study was to assess the additional value of genetic information in prognostic models in a hospital based cohort of patients who have suffered from cerebral ischemia of arterial origin (CIAO). We benefited from continuing efforts in the international stroke genetics community by incorporating results from the METASTROKE study.[8] With the observed effect estimates for selected SNPs (enriched for association with ischemic stroke), we calculated a genotype-based score for each individual in our cohort. Because the data from the GWAS were collected independently from our cohort, we were able to directly and rigorously assess the prognostic value of our prediction models.

## Methods

### Study design and patient population

The rationale of this study is described elsewhere in detail.[10] We collected data of patients with non-disabling cerebral ischemia of arterial origin, who were referred to the University Medical Center Utrecht, The Netherlands and were included in the SMART (Second Manifestations of Arterial disease) study, or the Utrecht Stroke Database (USDB). A detailed description of the SMART study was published previously.[11] Briefly, patients who gave their written informed consent underwent a standardised vascular screening programme, including a health questionnaire, laboratory assessment, and ultrasonography to investigate the prevalence of additional vascular diseases. Patients were followed up with bi-annual questionnaires. In the USDB extensive baseline data have been collected for consecutive patients visiting the University Medical Center Utrecht for TIA or stroke since 1991. Patient data was used anonymized in our analyses. Blood samples were taken from 1999 onwards and stored in the Neurology Blood Bank. Follow-up data were collected by contacting these patients or their general practitioners. Patients with non-atherosclerotic causes of cerebral ischemia or with potential source of embolism in the heart were excluded from this study. We therefore included only patients with cerebral ischemia of arterial origin. For the current study, the data of 1125 patients were available. These patients were included between April 1994 and May 2009.

### Outcomes

The primary outcome event was defined as a composite of the first occurrence of myocardial infarction, ischemic stroke or vascular death not due to hemorrhage. Secondary outcome event was recurrent ischemic stroke (Table 1). For potential outcome events reported by the patient we retrieved hospital discharge letters and the results of relevant laboratory and radiology examinations. Three members of the SMART Outcome Committee independently audited events on basis of available information. This committee consisted of physicians from different departments. In case of disagreement, consensus was reached by consulting other members of the Outcome Committee. Potential outcomes in patients included from the USDB were audited similarly.

**Table 1:** Definitions of outcome events.

*Event*	*Definition*
Ischemic stroke	Relevant clinical features that caused an increase in impairment of at least one grade on the modified Rankin scale^21^ associated with a relevant infarction on a repeat brain scan.
Myocardial infarction	At least two of the following criteria:
	1: Chest pain >20 min, not disappearing after administration of nitrates
	2: ST elevation >1 mm in two following leads or a left bundle branch block
	3:CK elevation of at least two times its normal value and an MB fraction >5% of total CK
Vascular death: not due to hemorrhage	Sudden death: unexpected coronary death occurring within 1 h after onset of symptoms or within 24 h given convincing circumstantial evidence.
	Terminal heart failure
	Fatal myocardial infarction or ischemic stroke

Primary outcome was defined as all fatal and non-fatal ischemic events. Secondary outcome was ischemic stroke separately.

### Genotyping

DNA samples available from both studies and stored in a -70 degrees Celsius freezer were transported to the Genetic Laboratory of the Department of Internal Medicine, Erasmus MC, Rotterdam, The Netherlands, where the samples were genotyped with the Illumina Immunochip.[12] The goal of the Immunochip was to provide a cost-effective genotyping platform for deep follow-up replication studies. It includes about 200,000 SNPs, selected in part on the basis of association results (low p-values) from a wide range of GWAS of immune-related diseases as well as the diseases covered by the second round of the Wellcome Trust Case Control Consortium (WTCCC-2), including ischemic stroke (http://www.wtccc.org.uk/ccc2/wtccc2_studies.shtml). About 2500 SNPs from an early analysis of the ischemic stroke GWAS by WTCCC-2 were contributed to the Immunochip design. We used these specific SNPs for our further analyses.

Quality control steps consisted of filtering of SNPs and individuals with >5% missing data, followed by filtering of SNPs with a minor allele frequency (MAF) <1% or deviation from Hardy-Weinberg equilibrium (HWE; p < 10^−6^). We then used individual-pairwise identity-by-state estimates to remove (unknown) related and potentially contaminated samples. We pruned SNPs by their pairwise linkage disequilibrium to arrive at a set of independent SNPs. Data processing and quality control filtering were performed in PLINK.[13] Principal components analysis was used to check genetic clustering of all individuals against reference individuals from the HapMap.[14]

### Individual genetic risk score

For each individual patient we calculated a genetic risk score on the basis of the genotypes of 1501 QC-passing SNPs and their effect sizes that were independently obtained from the GWAS of ischemic stroke led by the METASTROKE consortium.[8] Using PLINK, we calculated the genetic risk score as the sum of the ln(OR) multiplied by the number of risk alleles carried for each SNP considered in a given individual, following a method described elsewhere. [15]

### Statistical analysis

The prediction models were built with Cox regression analyses in SPSS. In univariable analysis we calculated the hazard ratios and corresponding 95% confidence intervals (CI) of different stroke risk factors. The different clinical variables included were adapted from a previous study, in which they determined long term survival and recurrent vascular event risk in patients with cerebral ischemia.[16] We then constructed the prediction model, in which we sequentially entered variables from the patients’ history until no remaining candidate variable had a significance level of <0.10 and into which we forced the genetic risk score based on METASTROKE effect estimates. Next we constructed receiver-operator characteristics (ROC) curves to compare the discriminatory performance with its area under the curve (AUC) of the model with and without genetic information. All analyses were done for the primary and secondary outcome.

## Results

### Baseline

Data were available of 1125 patients presenting with transient or non-disabling manifestations of cerebral or retinal ischemia and with information on genetic variants. Of these, 105 patients had to be excluded from further analysis because of genotyping quality concerns or unexpected relatedness between patients. These patients were similar to the remaining patients with respect to baseline characteristics (Table 2).

**Table 2:** Baseline characteristics.

	*Included patients*		*Excluded patients*
	1020		105
	Patients with primary outcome	Patients without primary outcome	
	198	822	
Age (years) (mean, SD)	66 (10)	62 (11)	63 (11)
Male sex	150 (76%)	518 (63%)	68 (65%)
Qualifying diagnosis			
TIA	75 (38%)	283 (34%)	30 (28%)
Stroke	93 (47%)	451 (55%)	66 (62%)
Transient Monocular Blindness	24 (12%)	79 (10%)	6 (6%)
Retinal infarction	6 (3%)	9 (1%)	3 (3%)
Subtype Qualifying diagnosis			
Large vessel disease	141 (71%)	552 (67%)	57 (54%)
Small vessel disease	57 (29%)	270 (33%)	48 (46%)
History			
Stroke	53 (27%)	161 (20%)	17 (16%)
Carotid surgery	14 (7%)	21 (3%)	3 (3%)
Myocardial infarction	37(19%)	80 (10%)	9 (9%)
Vascular surgery	51 (26%)	119 (15%)	16 (15%)
Hypertension	99 (50%)	391 (48%)	50 (47%)
Diabetes Mellitus	37 (19%)	106 (13%)	19 (18%)
Hyperlipidemia	60 (30%)	280 (34%)	36 (34%)
Cigarette smoking			
Currently	35 (18%)	198 (24%)	28 (26%)
Never or ever	159 (81%)	559 (73%)	71 (67%)
Blood pressure (mm Hg)			
Systolic (mean, SD)	157 (28)	149 (25)	153 (28)
Diastolic (mean, SD)	85 (14)	84 (13)	84 (14)
Glucose (mmol/L) (mean, SD)	6.6 (2.4)	6.4 (1.9)	6.5 (2.0)

* Patients were excluded because of quality concerns

At baseline patients had a mean age of 63 years and 66% was male (Table 2). The index event was a minor stroke in 53% of the patients, 35% had had a TIA and 12% suffered from an ischemic ocular event. Almost 50% of the patients had hypertension and 20% suffered from an earlier stroke. Patients with a recurrent ischemic vascular event had overall more risk factors for vascular disease than patients who had no recurrence.

### Follow up

The median follow-up time was 6.5 years (6630 person-years). The follow-up was complete in 99.5% of the patients. The annual risk of ischemic events was 3.0% (198 events). Half of the events were a fatal or non-fatal ischemic stroke (98 patients), 65 events concerned fatal or non-fatal myocardial infarction and the remaining 35 were other vascular death.

### Prognostic value of classical vascular risk factors

**Fig 1:**
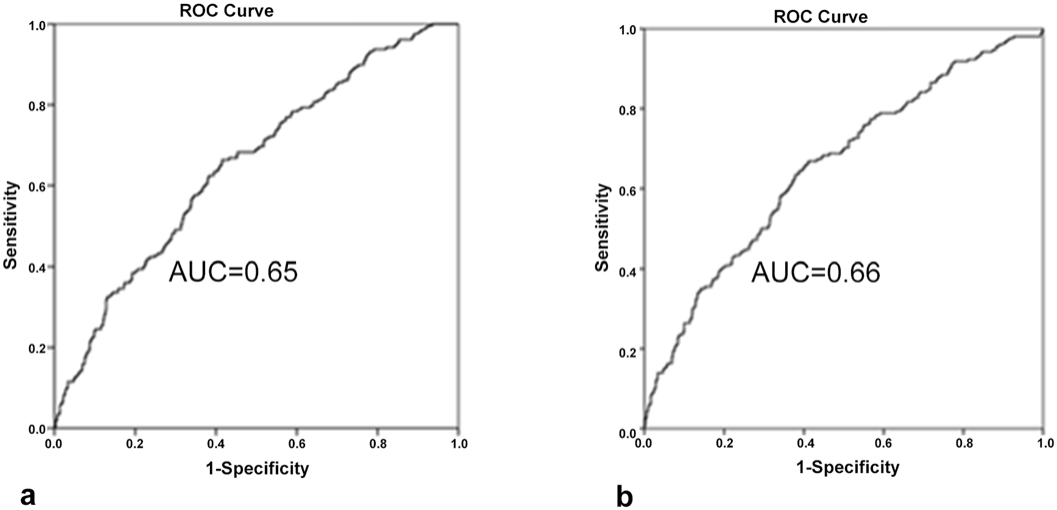
ROC curves for the primary outcome. (a) based on classical risk factors only; (b) based on classical risk factors plus the genetic risk score.

The different AUC-ROC values for the primary and secondary outcomes are displayed in Table 3 and for the primary outcome the ROC-curves are displayed in Fig 1. For the primary outcome the prognostic model consisted of the variables age, sex, myocardial infarction, intermittent claudication, diabetes mellitus and vascular surgery. The AUC-ROC was 0.65 (95% CI 0.61–0.69). For the secondary outcome, the AUC-ROC was 0.60 (95% CI 0.54–0.66), this included the vascular risk factors age, myocardial infarction, and hypertension.

**Table 3:** Cox proportional hazard models and AUC-ROC.

	*M1*		*M2*	
Indicator	HR	95% CI	HR	95% CI
**Demographic characteristics**				
Male	**1.47**	**1.06–2.04**	1.35	0.86–2.11
Age	**1.05**	**1.03–1.06**	**1.02**	**1.00–1.04**
**History**				
Myocardial infarction	**1.78**	**1.25–2.55**	**1.72**	**1.03–2.87**
Hypertension	1.08	0.82–1.43	**1.56**	**1.05–2.33**
Intermittent Claudication	**1.76**	**1.14–2.72**	1.55	0.80–2.97
Diabetes Mellitus	**1.37**	**0.96–1.97**	1.34	0.81–2.24
Vascular surgery	**1.79**	**1.30–2.46**	1.50	0.93–2.40
Genetic risk score	**1.13**	**0.92–1.38**	**1.17**	**0.91–1.50**
**AUC-ROC**				
Only classical risk factors	0.65	0.61–0.69	0.60	0.54–0.65
Plus genetic risk core	0.66	0.61–0.70	0.60	0.54–0.66

Table displays univariable analyses of risk factors for vascular disease for different endpoints. The bold numbers are accounted in the multivariable model with and without genetic risk scores. AUC-ROC = Area Under Curve of the Receiver Operating Characteristics Curve. M1 = primary outcome. M2 = secondary outcome, ischemic stroke

### Prognostic value of classical risk factors and genetic risk score

The vascular risk factors used in these models remained the same as the ones used in the first model. After addition of the genetic risk score the AUC-ROC for the primary outcome was essentially identical to that of the model with only classical vascular risk factors. For cerebral ischemia the AUC-ROC showed similar patterns (Table 3).

## Discussion

In our cohort of patients with cerebral ischemia of arterial origin we found no additional value of genetic information in the prediction of new ischemic events including ischaemic stroke. The overall prognostic performance of the known classical risk factors for vascular diseases is poor (AUC-ROC 0.65; 95%CI 0.61–0.69), consistent with similar prediction models described in the literature.[3] The inclusion of a genetic risk score based on SNPs that could be associated with ischemic stroke did not result in a prognostic model that improved risk stratification.

As far as we know, our study is the first to characterize the prognostic value of genetic information in the assessment of future risk for vascular events after cerebral ischaemia. Our cohort comprises well phenotyped ischemic stroke patients from a single medical center with uniform follow-up data. The origin from a single hospital could also be seen as a limitation with respect to the generalisability of the results. However, we feel that our cohort is representative since the results for classical risk factors for atherosclerosis affecting prognosis, like previous stroke, diabetes mellitus or hypertension, are consistent with other studies.

The sample size is a major limitation. We had to drop almost 10% of our population due to quality concerns of the genetic data. These patients, however, had similar clinical characteristics as the remaining patients. Furthermore we included only non-disabled patients on the (predefined) basis that this patient group is the one most relevant, with the highest chance of survival and long-term prospects for recovery, and therefore represents the main focus of our study.

The selection of SNPs and the nature of the external training data set could be seen as a limitation of our study. We were not aware of another dataset on patients with cerebral ischemia of arterial origin followed prospectively for the occurrence of ischemic events or bleedings in whom also genetic data were available (which we could have used as an alternative training data set). We therefore decided to use the observed effect estimates from the METASTROKE study, even though this study focused on prevalent (ischemic) stroke cases in the population instead of the occurrence of recurrent vascular events. It is certainly possible that genes influencing the risk of a first cerebral ischemic event differ from those genes associated with long-term prognosis after an ischemic event. In addition, we only used the effect estimates for all types of ischemic stroke instead of those for small and large vessel stroke subtypes, because our sample was too small to look into individual subtypes of stroke.

Our results illustrate the complexity of finding susceptibility alleles for a clinical phenotype that is so complex and heterogeneous such as ischemic stroke. To date, only a handful of robust SNP associations have been identified for cardioembolic stroke (*PITX2*, *ZFHX3*), large-artery atherosclerotic stroke (9p21, *HDAC9*), and more recently, for all subtypes of stroke (12q24). [17–18]The approach to test the collective effects of many common variants simultaneously has been successfully applied in complex traits such as schizophrenia and bipolar disorder [15]

We were only informed about the use of medication at baseline of the patients in this study. Platelet function, response to pharmacotherapy and genetic factors may be associated with outcome and recurrent risk. The debate about the role of aspirin resistance and the way patient tailored treatment strategies could be implemented in daily practice is still ongoing.[19–21] The same is true for other antithrombotic treatment strategies as, for example clopidogrel, for which genetic association with its metabolism is reasonably well understood.[22]

Future stroke genetic studies might result in better risk prediction for different stroke subtypes and recurrent events. It remains unresolved today, however, to what extent genetic variants of modest effect (even if many more are identified as a result of larger discovery efforts) could contribute to prognostic models for such complex phenotypes as stroke above and beyond family history and traditional risk factors.

### Integrity of research and reporting

The Ethics Committee of the hospital approved both studies (SMART and USDB) from which we used the data. All patients included in these studies gave written informed consent prior to inclusion.

The authors had full access to the data and take responsibility for its integrity. All authors have read and agree to the manuscript as written. The authors declare that they have no conflict of interest.

The data of this study will be made available upon request
